# Immunogenicity of a Virus-Like-Particle Vaccine Containing Multiple Antigenic Epitopes of *Toxoplasma gondii* Against Acute and Chronic Toxoplasmosis in Mice

**DOI:** 10.3389/fimmu.2019.00592

**Published:** 2019-03-29

**Authors:** Jingjing Guo, Aihua Zhou, Xiahui Sun, Wenchao Sha, Kang Ai, Ge Pan, Chunxue Zhou, Huaiyu Zhou, Hua Cong, Shenyi He

**Affiliations:** ^1^Department of Parasitology, School of Basic Medical Sciences, Shandong University, Jinan, China; ^2^Department of Pediatrics, Provincial Hospital Affiliated to Shandong University, School of Medicine, Shandong University, Jinan, China

**Keywords:** *Toxoplasma gondii*, virus-like particles, HBc, multiple antigenic epitopes, vaccine

## Abstract

There is no effective protective vaccine against human toxoplasmosis, which is a potential threat to nearly a third of the world population. Vaccines based on virus-like particles (VLPs) have been highly successful in humans for many years, but have rarely been applied against *Toxoplasma gondii* infection. In this study, we inserted a B cell epitope (SAG1_82−102_ or SAG1_301−320_), a CD8^+^ cell epitope (HF10 or ROP7), and a CD4^+^ cell epitope (AS15) of *T. gondii* into a truncated HBc_Δ_(amino acids1–149) particle to construct four chimeric VLP vaccine formulations, i.e., HBc_ΔH82_, HBc_ΔH301_, HBc_Δ R82_, and HBc_Δ R301_. When these chimeric HBc particles were expressed in *Escherichia coli*, they showed icosahedral morphology similar to that of the original VLPs and were evaluated as vaccine formulations against acute and chronic toxoplasmosis in a mouse model (BALB/c mice (H-2^d^). All these chimeric HBc VLPs induced strong humoral and cellular immune responses with high IgG antibody titers and interferon(IFN)-γ production. Only the mice immunized with HBc_ΔH82_ showed prolonged survival time (15.6 ± 3.8 vs. 5.6 ± 0.8 days) against acute infection with RH tachyzoites and decrease in brain parasite load (1,454 ± 239 vs. 2,091 ± 263) against chronic infection with Prugniuad cysts, as compared to the findings for the control group. These findings suggest that HBc VLPs would act as an effective carrier for delivering effective multiple antigenic epitopes and would be beneficial for developing a safe and long-acting vaccine against toxoplasmosis.

## Introdction

*Toxoplasma gondii*, an obligate intracellular protozoan, is the causative agent of toxoplasmosis. The disease is a severe health threat for fetuses, newborns, and immunocompromised individuals (1). Current treatments cannot completely control this disease because of the ineffectiveness of drugs in killing the latent cyst form of the parasite (2). Developing effective vaccines could help prevent *T. gondii* infection (3). However, a licensed vaccine for human use is not available.

Virus-like particles (VLPs) are diverse nanoparticles (size, 20–100 nm) that are formed by structural viral proteins, such as capsids, and can self-assemble. They resemble viruses but are noninfectious because they lack viral genetic materials (4, 5). VLPs mimic the 3D conformation of native viruses and display a high density of repetitive effective antigenic epitopes on their surface, which stimulate the desired humoral and cellular responses in humans (6). In recent years, VLPs have provided an excellent option as vectors for producing vaccines against infectious diseases; four VLP vaccines are available commercially (7). However, not much research has been performed on VLP vaccines against *T. gondii*, and more work is required to fill the gaps in this promising field.

Hepatitis B virus (HBV) core antigen (HBc) has been extensively used as a VLP platform for many years (6). It is highly expressed in many recombinant gene expression systems, including prokaryotic expression systems, and is easy to self-assemble *in vitro* (8). It can greatly improve the immunogenicity of foreign antigenic epitopes presented on its surface, especially those inserted into the major immunedominant region (MIR) of the capsid located at the tips of the surface “spikes” (9). Various foreign antigens from bacteria, viruses, and protozoa have been genetically inserted into the particles, and some of these HBc-antigen fusions have reached the clinical testing stage (6, 10).

For vaccines to be effective against toxoplasmosis, they should include antigen epitopes that can elicit a protective Th1 immune response, characterized by the generation of long-lived CD8^+^ T cells that can secrete interferon (IFN)-γ and develop cytotoxic activity against infected cells (11). CD8^+^ cytotoxic T lymphocyte (CTL)-mediated resistance to *T. gondii* cysts in the brain is absolutely correlated with the major histocompatibility complex (MHC) class I *L*^*d*^ allele in mice (12, 13). The secreted proteins of *T. gondii*, such as dense granules (GRAs) and rhoptry proteins (ROPs), are antigens that are recognized by murine T lymphocytes and are vaccine candidates against toxoplasmosis (11). The peptide (HPGSVNEFDF) (HF10), derived from GRA6, and the peptide (IPAAAGRFF), derived from ROP7, are the protective immunodominant *L*^*d*^-restricted epitopes in mice (1, 14). Antibodies also play an important role in host immunity against *T. gondii*; they can directly block tachyzoites and impair their attachment to host cells. SAG1 protein is the major immunogenic surface antigen that is involved in attachment to host cells during *T. gondii* invasion, which makes it a suitable source of B cell epitopes (15). SAG1_82−102_ is a highly immunogenic conformational B cell epitope but was not considered as a vaccine candidate because of the lack of a suitable carrier to maintain the native spatial conformation until date (16, 17). SAG1_301−320_ is a linear B cell epitope that can protect mice against a lethal challenge and is strongly recognized by sera from toxoplasmosis patients (18, 19). Moreover, CD4^+^ T cells constitute an important component of the immune response to *T. gondii*, and AS15 is a CD4^+^ T cell-stimulating peptide that can confer protection against toxoplasmosis (20). Most of these molecules are conserved between type I and type II strains of *T. gondii*. Although all three strains of the parasite have been isolated from humans, type I and type II strains are more often associated with human disease and the type III strain seems to be more common in animals (21, 22).

In this study, we designed four HBc VLP vaccine formulations, i.e., HBc_ΔH82_, HBc_ΔH301_, HBc_ΔR82_, and HBc_ΔR301_, comprising a B cell epitope (SAG1_82−102_ or SAG1_301−320_), a CD8^+^ cell epitope (HF10 or ROP7), and a CD4^+^ cell epitope (AS15). The efficacy of these vaccines in eliciting humoral and cellular immune responses in BALB/c mice was examined. Immunization of these mice activated CD8^+^ T cells to produce IFN-γ, activated B cells to produce antibodies, and protected against subsequent challenge with type I and type II strains of *T. gondii*. Our results indicated that a VLP-based immunization with complex and heterogeneous antigenic epitopes is a promising vaccine approach to protect against toxoplasmosis.

## Materials and Methods

### Mice

Female 6–8 week old BALB/c mice (H-2^d^) were purchased from Shandong University Laboratory Animal Center (Jinan, China). All the mice were bred and maintained in small groups inside separate cages under a 12-h light/dark cycle (lights switched on at 7:00 am). Water and food were available *ad libitum* to all the mice throughout. The protocol was approved by the Institutional Animal Care and Use Committee of Shandong University (Contract LL201602044). All efforts were made to minimize animal suffering and the number of mice used.

### Parasites

The RH strain, a high-virulence strain of *T. gondii* (type I), was kindly provided by Professor Jilong Shen (Anhui Medical University, Hefei, China). Cultures of RH tachyzoites were propagated in human foreskin fibroblast (HFF) cells in Dulbecco's modified Eagle's medium (DMEM; Sigma-Aldrich, St.Louis, MO) supplemented with 10% (vol/vol) fetal bovine serum (FBS; Clark Biosciences, Seabrook, MD), as previously described (23). The Prugniuad (Pru) strain, a low-virulence strain of *T. gondii* (type II), was a kind gift from Professor Xingquan Zhu (Lanzhou Veterinary Research Institute, China). The cysts of the Pru strain were maintained in Kunming mice by oral passage of infectious cysts in the mice, as previously described (24).

### Construction of Prokaryotic Expression Plasmids

**Figure 1:**
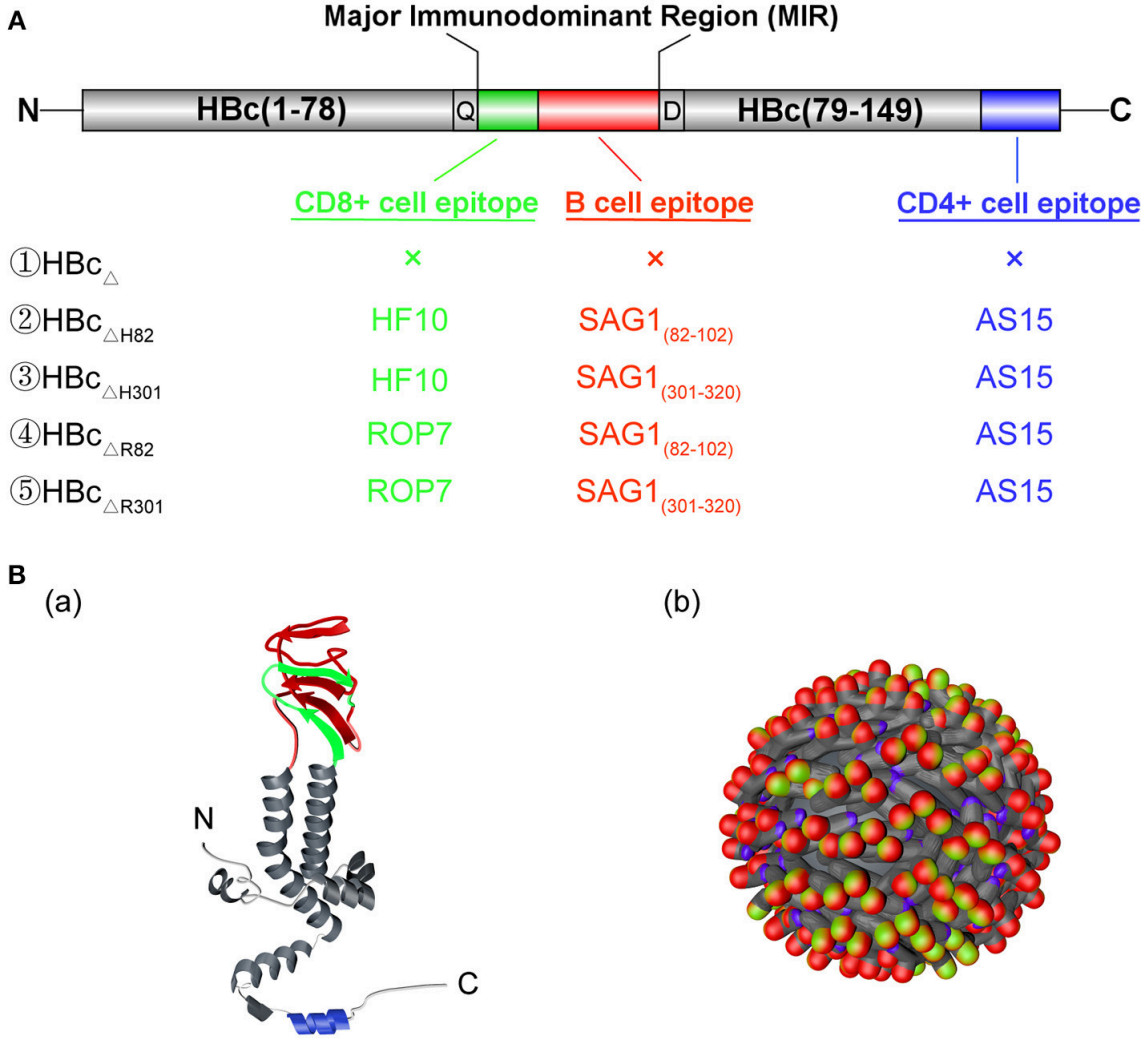
Structure of the chimeric HBc virus-like particles (VLPs). **(A)** Gene structure of the chimeric HBc VLPs. HBc_Δ_ represents the truncated HBc particles (aa 1–149). A CD8^+^ T cell epitope (HF10 or ROP7; green) and a B cell epitope (SAG1_82−102_ or SAG1_301−320_; red) of *Toxoplasma gondii* were inserted between aa78 and 79 of HBc_Δ_ particles (MIR), with a Gln(Q) and Asp(D) linker at both ends. Futhermore, a CD4^+^ cell epitope (AS15) (blue) of *T. gondii* was fused into the C terminal of HBc_Δ_ particles. **(B)** The monomer (a) and polymer (b) structures of the chimeric HBc VLPs. The inserted CD8^+^ T cell, CD4^+^ T cell, and B cell epitopes are shown in green, blue, and red, respectively.

When used as a carrier protein to present antigens, HBc particles have historically been truncated at amino acid 149 to avoid incorporation of host RNA by the C-terminal tail [amino acids (aa) 150−183], which contains the RNA/DNA-binding site of the viral capsid (10). The truncated HBc particles have been represented by HBc_Δ_ in this study. A CD8^+^ cell epitope (HF10 or ROP7) and a B cell epitope (SAG1_82−102_ or SAG1_301−320_) of *T. gondii* were inserted between amino acids 78 and 79 of HBc_Δ_ particles, with a Gln (Q) and Asp (D) linker at both ends (Figure 1A). A CD4^+^ cell epitope (AS15) was fused into the C terminal of HBc_Δ_ particles. The final constructs were named HBc_Δ_, HBc_ΔH82_, HBc_ΔH301_, HBc_ΔR82_ and HBc_ΔR301_. The amino acid sequences of the inserted B, CD8^+^, and CD4^+^ cell epitopes are provided in Table 1. After addition of a C-terminal His6 tag for purification and 5′ NdeI and 3′ XhoI overhangs for cloning into the expression plasmid as previously described (25), all the gene segments of the chimeric HBc VLPs were synthesized by Genewiz Biotech Ltd (Suzhou, China) and then cloned into the pET-30a(+) plasmid (Novagen, Madison, WI). Five recombinant plasmids, i.e., pET-30a(+)/HBc_Δ_, HBc_Δ*H*82_, HBc_Δ*H*301_, HBc_Δ*R*82_, and HBc_ΔR301_, were verified by DNA sequencing. The recombinant plasmid pET-30a(+)/HBc_Δ_ was used as a negative control.

**Table 1:** Identified B cell and T cell epitopes of *T. gondii* used in this study.

**Epitope**	**Type**	**Conformational/liner**	**Amino acid sequence**
1. SAG1(82-102)	B cell epitope	Conformational	CPKTALTEPPTLAYSPNRQIC
2. SAG1(301-320)	B cell epitope	Liner	FAGAAGSAKSAAGTASHVSI
3. HF10(GRA6)	CD8^+^ cell epitope	Liner	HPGSVNEFDF
4. ROP7	CD8^+^cell epitope	Liner	IPAAAGRFF
5. AS15	CD4^+^cell epitope	Liner	AVEIHRPVPGTAPPS

### Expression of Chimeric HBc VLPs

The procedure for expression of the chimeric proteins was performed as previously described (26), with minor modifications. Briefly, *Escherichia coli* BL21(DE3) (Novagen) cells transformed with the recombinant plasmids, i.e., pET-30a (+)/HBc_Δ_, HBc_ΔH82_, HBc_ΔH301_, HBc_ΔR82_, and HBc_ΔR301_, were grown on Luria-Bertani (LB) agar plates containing kanamycin (50 μg/ml). Individual colonies were cultured at 37°C with shaking at 220 rpm in 3 ml LB medium supplemented with kanamycin (50 μg/ml). On the next day, the night cultures were transferred to 500 ml fresh LB medium with kanamycin for amplification. When the optical density (OD_600_) of the cultures reached 0.6–0.8, expression of the chimeric proteins was induced by adding 0.5 mM isopropyl-β-D-thiogalactopyranoside(IPTG; Sigma-Aldrich). The cultures were grown for an additional 16–18 h at 11°C and harvested by centrifugation at 4,000 × g for 20 min at 4°C. Resuspended cell pellets were disrupted by sonication, subjected to 12% sodium dodecyl sulfate (SDS)–polyacrylamide gel electrophoresis (PAGE), and stained with Coomassie Blue to analyse chimeric VLPs expression.

### Purification of Chimeric HBc VLPs

The recombinant proteins were purified as described in a previous study (27). The cell pellets of IPTG-induced *E. coli* transformants were collected and resuspended in lysis buffer (20 mM Tris-HCl, 1 mM phenylmethylsulfonyl fluoride [PMSF], and bacterial protease-inhibitor cocktail, pH 8.0). These cells were broken up and the chimeric proteins were released by sonication at 400 W for 4 s, performed at 8-s intervals for a total of 20 min. Most of the target proteins existed in the form of inclusion bodies in the *E. coli* expression system. After the inclusion bodies were collected by centrifugation at 10,000 × g for 20 min at 4°C, they were washed three times with wash buffer (20 mM Tris, 1 mM EDTA, 2 M urea, 1 M NaCl, 1% Triton X-100, pH 8.0) and dissolved in lysis buffer (20 mM Tris, 5 mM DTT, 8 M urea, pH 8.0) in a certain ratio overnight. Then, the supernatant was added gradually to the buffer (20 mM Tris-HCl, 100 mM NaCl, pH 8.0), stirred slowly, and gradient-diluted. Then the protein solution was dialyzed against the same buffer overnight in the dialysis bag (Merck Millipore, Billerica, MA). After the supernatant was recovered, His-tagged chimeric HBc VLPs were purified using Ni2^+^ iminodiacetic acid (IDA) affinity chromatography gel (Novagen), following the procedure recommended by the manufacturer.

To identify the chimeric proteins, western blot analyses were performed using an anti-His Tag monoclonal antibody (Abcam, Cambridge, MA), followed by detection with horseradish peroxidase (HRP) Affinipure goat anti-mouse IgG (H+L) (ZSGB-BIO, Beijing, China). HBc_Δ_ protein was used as a negative control in all three experiments. Endotoxin contamination in the chimeric HBc VLPs was analyzed using *Limulus* amebocyte lysate(LAL) (ZhanJiang A&C Biological Ltd, Shanghai, China) (28).

Four multiepitope peptides, i.e., H82, H301, R82, and R301, containing a CD8^+^ cell epitope (HF10 or ROP7), a B cell epitope (SAG1_82−102_ or SAG1_301−320_), and a CD4^+^ cell epitope (AS15) were synthesized (>95% purity) by Synpeptide Co., Ltd. (Shanghai, China) and stored at −80°C until use.

### Electron Microscopy

The purified chimeric HBc VLPs, i.e., HBc_Δ_, HBc_ΔH82_, HBc_ΔH301_, HBc_ΔR82_, and HBc_ΔR301_, were adsorbed onto carbon-formvar-coated copper grids (200 mesh) and negatively stained with 1% phosphotungstic acid as previously described (29). Three random fields were observed using a transmission electron microscope (TEM; JEM-1011) at an accelerating voltage of 100 kV with 120,000 × magnification. HBc_Δ_ particle was used as a negative control.

### Immunization and Challenge in Mice

The mice were segregated into groups of 23 each and immunized subcutaneously with 50 μg of a recombinant protein, i.e., HBc_Δ_, H82, HBc_ΔH82_, H301, HBc_ΔH301_, R82, HBc_ΔR82_, R301, or HBc_ΔR301_) or 50 μl PBS (blank control) on Days 0, 14, and 28 as previously described (30). For monitoring of the humoral response, serum samples were collected by retro-orbital bleeding at 0, 2, 4, and 6 weeks after the first immunization and were stored at −80°C until analysis. For monitoring of the cellular response, lymphocyte proliferation and cytokine production tests were performed. At 2 weeks after the last immunization, three mice per group were sacrificed and their spleens were obtained (**Figure 4A**).

To investigate the immune protection of the chimeric HBc VLPs against acute *T. gondii* infection, 10 mice per group were infected intraperitoneally with a lethal dose (1 × 10^3^ tachyzoites) of the RH strain and were monitored over 20 days as performed previously (58). To evaluate the effect of vaccination in mice with chronic toxoplasmosis, the remaining 10 mice in each group were challenged orally with a sublethal dose (30 cysts) of the Pru strain as previously described (31) (**Figure 4A**). All the mice were euthanized at 45 days after the challenge and their brains were removed and homogenized in 1 ml PBS. The *T. gondii* cyst burden in the mouse brains was confirmed by analyzing 10 μl samples of each cerebral homogenate with a light microscope as previously described (58).

### Evaluation of the Humoral Response

The *T. gondii*-specific IgG, IgG1 and IgG2a antibody levels in the serum samples were measured using enzyme-linked immunosorbent assay (ELISA) as previously described (30). Briefly, 50 μg of the recombinant proteins, i.e., HBc_Δ_, H82, HBc_ΔH82_, H301, HBc_ΔH301_, R82, HBc_ΔR82_, R301, and HBc_ΔR301_, or 50 μl PBS (blank control) was adsorbed overnight onto 96-well plates (Corning Incorporated, Corning, NY) at 4°C in 50 mM carbonate buffer (pH 9.6). After blocking with 1% low fat milk in phosphate-buffered saline (PBS) containing 0.05% Tween 20 (PBST) for 1 h at 37°C, the mouse serum was diluted in PBS (1:25) and incubated at 37°C for 1 h. After washing three times with PBST, the anti-mouse-IgG, IgG1 or IgG2a HRP-conjugated antibodies (Sigma-Aldrich) were added. Peroxidase activity was detected using 10 mg/ml 3,3′,5,5′-tetramethylbenzidine (TMB; Sigma-Aldrich) and stopped by adding 50 μl 2M H_2_SO_4_. The results were recorded as the absorbance at 450 nm and detected with a Thermo Scientific Multiskan (Thermo, Waltham, MA) FC Microplate Photometer. All assays were performed in triplicate.

### Evaluation of the Cellular Response

The percentages of the CD4^+^ and CD8^+^ T lymphocytes subsets of immunized mice were analyzed by flow cytometry as previously described (32). Briefly, 2 weeks after the last immunization, splenocyte suspensions were prepared from three immunized mice per group by pushing the spleens through a wire mesh. After the erythrocytes were lysed, the splenocytes were suspended in Roswell Park Memorial Institute (RPMI)-1640 medium supplemented with 10%FBS. The cells were plated in 96-well plates in triplicate at a density of 2 × 10^5^ cells/well and were stained with anti-mouse CD3-APC (eBiosciences, San Diego, CA), anti- mouse CD4-FITC (eBiosciencesA), and anti-mouse CD8-PE (eBiosciences) for 30 min at 4°C in the dark. All these cells were analyzed with a Cytoflex S Flow Cytometer (Beckman Coulter, USA), and the data were analyzed with the CytExpert software.

To determine the cytokine levels, splenocytes (2 × 10^6^ cells/ml) were stimulated with 10 μg/ml of the recombinant proteins, i.e., HBc_Δ_, H82, HBc_ΔH82_, H301, HBc_ΔH301_, R82, HBc_ΔR82_, R301, or HBc_ΔR301_, or 5μg/ml Con A (Sigma-Aldrich) (positive control), or 50 μl PBS (blank control), as previously described (33, 34). The cell-free supernatants from cultured splenocytes were collected and assayed for interleukin (IL)-2 and IL-4 activities at 24 h, for IL-10 activity at 72 h, and for IFN-γ activity at 96 h. The cytokine levels were measured using a commercial ELISA kit (eBioscience) according to the manufacturer's instructions. All assays were performed in triplicate.

## Statistical Analysis

Statistical analysis was performed using SPSS19.0 and GraphPad Prism 7.0. Differences between groups were analyzed using one-way analysis of variance (ANOVA). The survival percentage was analyzed with the Kaplan-Meier test, and survival curves were compared using the log-rank test. Data have been expressed in terms of mean ± standard deviation (SD) values. Values of *p* < 0.05 were considered statistically significant.

## Results

### Gene Engineering and Chimeric HBc VLP Production

**Figure 2:**
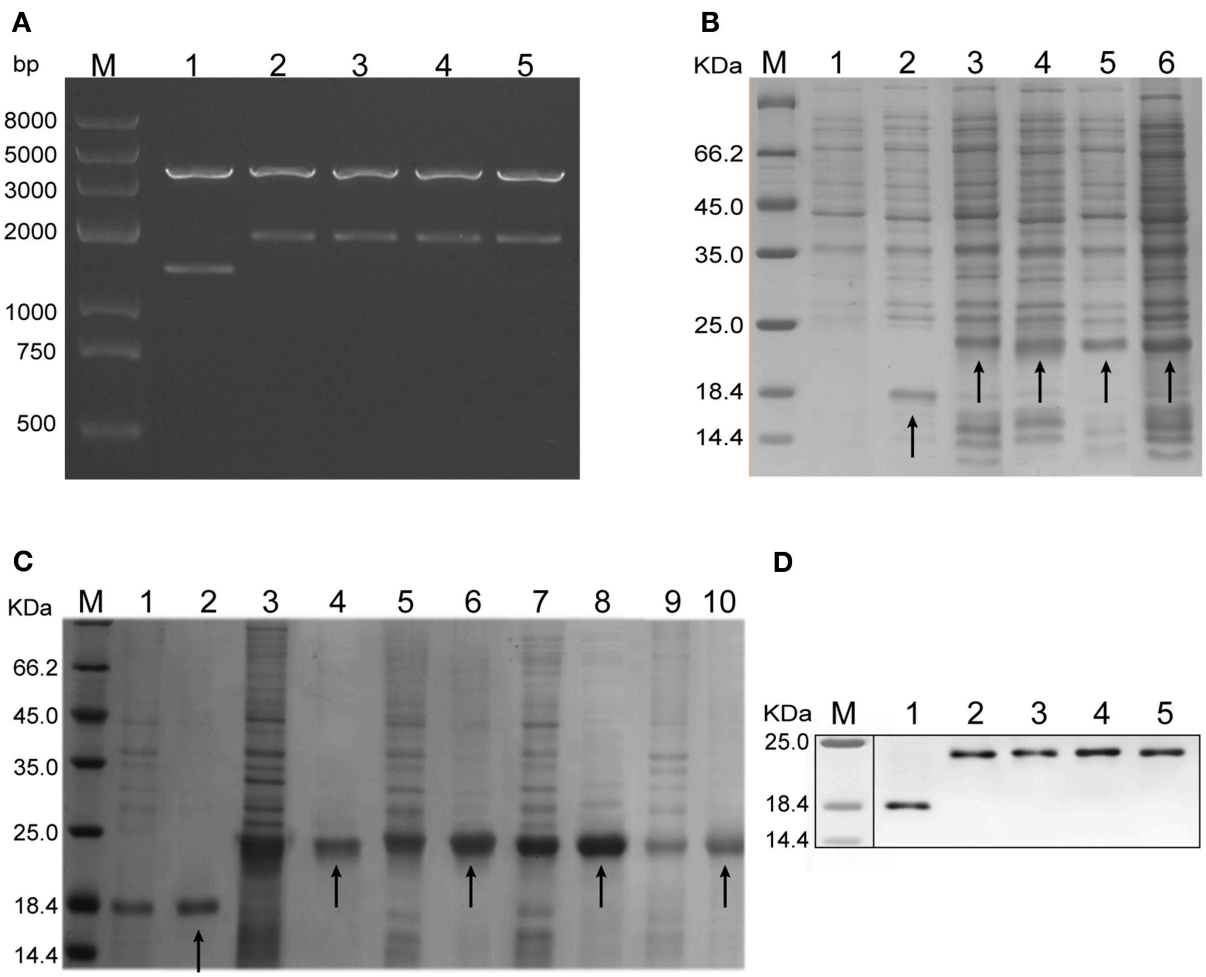
Plasmid identification and protein expression analysis. **(A)** Five recombinant plasmids, i.e., pET-30a (+) /HBc_Δ_, HBc_ΔH82_, HBc_ΔH301_, HBc_ΔR82_, and HBc_ΔR301_, were analyzed by agarose gel electrophoresis. They were digested into large fragments (4,193 bp) and small fragments (1,503, 2,097, 2,094, 2,094p, and 2,091bp, respectively) by two endonucleases (5'-*Apa*I and 3'-*Xho*I, respectively). Lane M: protein marker; Lanes 1–5: recombinant plasmids, i.e., pET-30a (+) /HBc_Δ_, HBc_ΔH82_, HBc_ΔH301_, HBc_ΔR82_, and HBc_ΔR301_. **(B)** Expression of the chimeric HBc VLPs in *Escherichia.coli* tested by sodium dodecyl sulfate (SDS)–polyacrylamide gel electrophoresis (PAGE) and Coomassie Blue staining. Lane M: protein marker; Lane 1: lysates of the pET-30a (+) transformant; Lanes 2-6: lysates of the pET-30a (+)/HBc_Δ_, HBc_ΔH82_, HBc_ΔH301_, HBc_ΔR82_, and HBc_ΔR301_ transformants. HBc_Δ_(18.3 KDa), HBc_ΔH82_ (23.2 KDa), HBc_ΔH301_(23.0 KDa), HBc_ΔR82_ (23.0 KDa),and HBc_ΔR301_ (22.8 KDa) proteins have been indicated by arrows. **(C)** Purification of the chimeric proteins analyzed by SDS-PAGE and Coomassie Blue staining. The efficiency of the protein purification and molecular mass of the chimeric HBc VLPs were ensured by using the unpurified proteins as templates. Lane M: protein marker; Lanes 1,3,5,7,and 9: unpurified HBc_Δ_, HBc_ΔH82_, HBc_ΔH301_, HBc_ΔR82_, and HBc_ΔR301_ protein, respectively; Lanes 2, 4, 6, 8, and 10: purified HBc_Δ_, HBc_ΔH82_, HBc_ΔH301_, HBc_ΔR82_, and HBc_ΔR301_ proteins, respectively (indicated by arrows). **(D)** Western blot analysis of the expressed proteins (using an anti-His tag mAb). Lane M: protein marker; Lanes 1–5: purified HBc_Δ_, HBc_ΔH82_, HBc_ΔH301_, HBc_ΔR82_, and HBc_ΔR301_ proteins.

The composition of the hybrid HBc genes, i.e., HBc_Δ_, HBc_ΔH82_, HBc_ΔH301_, HBc_ΔR82_, and HBc_ΔR301_, is shown in Figure 1A. Figure 1B shows the expected sites of appearance of these inserted epitopes on the surface of monomer (a) and polymer (b) of HBc VLPs. These genes were inserted into the plasmid pET-30a(+) to construct the recombinant plasmids pET-30a(+)/HBc_Δ_, HBc_ΔH82_, HBc_ΔH301_, HBc_ΔR82_, and HBc_ΔR301_. After digestion with restriction endonucleases, these plasmids were confirmed by agarose gel electrophoresis. The plasmids of chimeric HBc VLPs (HBc_ΔH82_, HBc_ΔH301_, HBc_ΔR82_, HBc_ΔR301_) were digested into large fragments (4193 bp) and small fragments (2097 bp, 2094 bp, 2094 bp, 2091 bp) by the 5'-*Apa*I and 3'-*Xho*I endonucleases, respectively, and the non-chimeric HBc particle (HBc_Δ_) was 4,193 bp and 1,503 bp correspondingly, which indicated that the recombinant plasmids contained inserts (Figure 2A).

After the chimeric HBc VLPs were expressed in *E. coli*, they were analyzed by SDS-PAGE and Coomassie Blue staining. Increased expression of the chimeric proteins HBc_Δ_ (18.3 KDa), HBc_ΔH82_ (23.2 KD), HBc_ΔH301_ (23.0 KDa), HBc_ΔR82_ (23.0 KDa) and HBc_ΔR301_ (22.8 KDa) was observed in the supernatants of cellular lysates obtained by sonication (Figure 2B). The chimeric proteins were purified using Ni2^+^ IDA affinity chromatography gels; the purified proteins showed a single band for each preparation with appropriate molecular masses (Figure 2C). These proteins were recognized by a His-tag mAb at the C terminus of the HBc particles by western blot analysis (Figure 2D). These results indicated that HBc VLPs carrying the multiepitopes of *T. gondii* were successfully expressed in a prokaryotic expression system. In addition, the LAL test showed 0.5 endotoxin units/μg for the chimeric HBc VLPs.

### Electron Microscopic Characterization of VLPs

**Figure 3:**
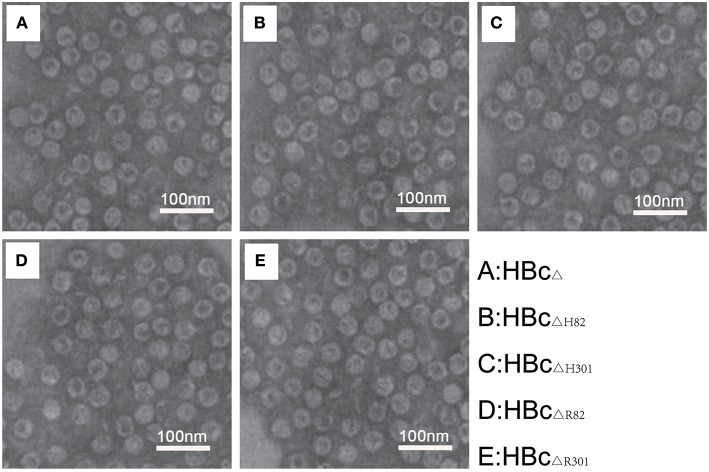
Electron microscopic characterization of VLPs. Transmission electron microphotographs of the chimeric HBc particles [HBc_Δ_ **(A)**, HBc_ΔH82_ **(B)**, HBc_ΔH301_ **(C)**, HBc_ΔR82_ **(D)**, and HBc_ΔR301_ **(E)**] showing regular morphology of the VLPs in each preparation. Images were generated using 1% phosphotungstic acid as a negative stain. Magnification 120, 000×. Scale bar, 100 nm.

A truncated version of HBc (aa 1–149), lacking a protamine-like domain (aa150–183), is known to be sufficient for self-assembly into capsid particles (35). TEM analysis with phosphotungstic acid negative staining showed that multiepitope immunogens of *T. gondii*-containing truncated HBc VLPs (HBc_ΔH82_, HBc_ΔH301_, HBc_ΔR82_, and HBc_ΔR301_) demonstrated good capability for self-assembly and had icosahedral morphology similar to the original corresponding non-chimeric VLPs (HBc_Δ_) (Figure 3). These particles were homogeneous in size.

### Humoral Immune Responses Induced by Vaccination

**Figure 4:**
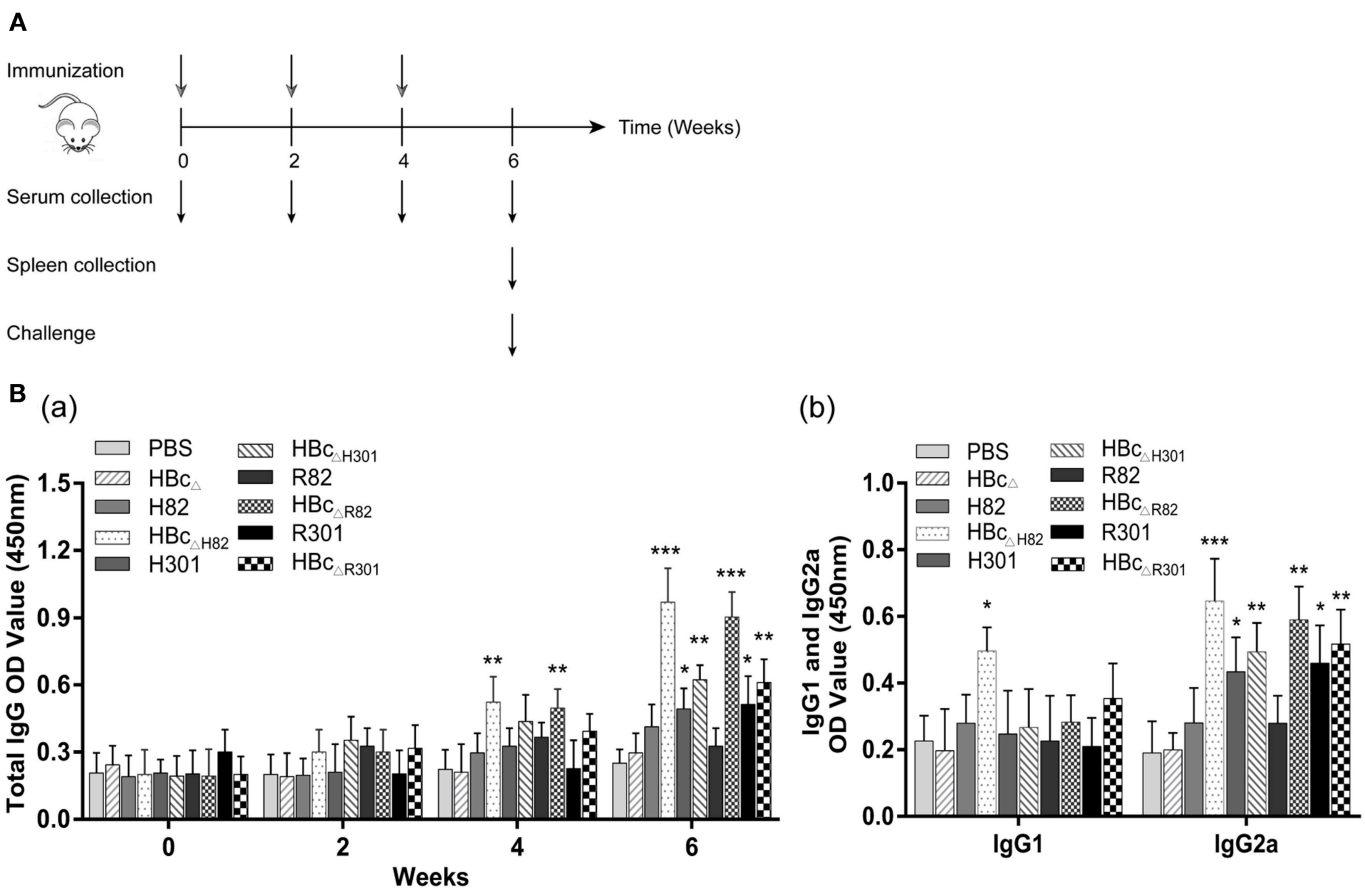
Immunization schedule and humoral immune response induced by vaccination. **(A)** Schematic diagram of the immunization protocol. BALB/c mice (23 mice/group) were immunized subcutaneously with 50 μg of the recombinant proteins (HBc_Δ_, H82, HBc_ΔH82_, H301, HBc_ΔH301_, R82, HBc_ΔR82_, R301, and HBc_ΔR301_) at 0,2, and 4 weeks after first immunization. Serum samples were collected at 0, 2, 4, and 6 weeks. Three mice per group were sacrificed and spleens were obtained 2 weeks after the last immunization. Then, 10 mice per group were infected intraperitoneally with 1 × 10^3^ tachyzoites of the RH strain, and the other 10 mice in each group were orally challenged with 30 cysts of the Pru strain. **(B)** Detection of the levels of *T. gondii*-specific IgG antibody (a) and IgG1 and IgG2a subclass antibodies (b) in the sera of the immunized mice by enzyme-linked immunosorbent assay (ELISA). Serum samples were collected from the mice by retro-orbital bleeding at 0, 2, 4, and 6 weeks after the first immunization. After stimulation with 10 μg/ml of the recombinant proteins, humoral immune responses were analyzed. Results have been expressed as the mean of OD_450_ ± SD values (*n* = 15) and are representative of at least three independent experiments. *p < 0.05, **p < 0.01, ***p < 0.001.

To detect the levels of anti-*T. gondii* antibodies, sera from the immunized mice were tested with ELISA. Total IgG antibodies were detectable as early as 4 weeks after the first vaccination in the mice immunized with HBc_ΔH82_ and HBc_ΔR82_ proteins compared with the mice immunized with PBS (Figure 4Ba). These two proteins induced the highest IgG antibody levels 6 weeks after the first vaccination in all vaccinated mice (*p* < 0.001). The IgG antibody levels in the sera of mice immunized with the recombinant proteins containing the SAG1_301−320_ epitope (H301, HBc_ΔH301_, R301, and HBc_ΔR301_) also increased. In contrast, the mice injected with HBc_Δ_, H82 and R82 did not have significant antibody titers as compared to those of the control group (PBS).

To determine whether a Th1 and/or a Th2 humoral response was induced by vaccination with the recombinant proteins, the levels of specific IgG1 and IgG2a subclasses were measured. Increased IgG2a production was observed in the sera of mice immunized with H301, HBc_ΔH301_, HBc_ΔR82_, R301, and HBc_ΔR301_ proteins, especially with HBc_ΔH82_ (*p* < 0.001; Figure 4Bb) compared with the control group (PBS). Only the mice vaccinated with the HBc_ΔH82_ protein showed increased IgG1 titers. These results indicated that all the recombinant proteins containing the linear B cell epitope SAG1_301−320_, i.e., H301, HBc_ΔH301_, R301, and HBc_ΔR301_, elicited more anti-*T. gondii* antibodies than the control group (PBS) did in the immunized mice. However, for the conformational B cell epitope SAG1_82−102_, the strong IgG antibody response was induced only after it was loaded onto the HBc VLP platform (HBc_ΔH82_ and HBc_ΔR82_) in mice, which can maintain its natural conformation. This specific immune response was of the Th1 type.

### Cellular Immune Responses Induced by Vaccination

**Figure 5:**
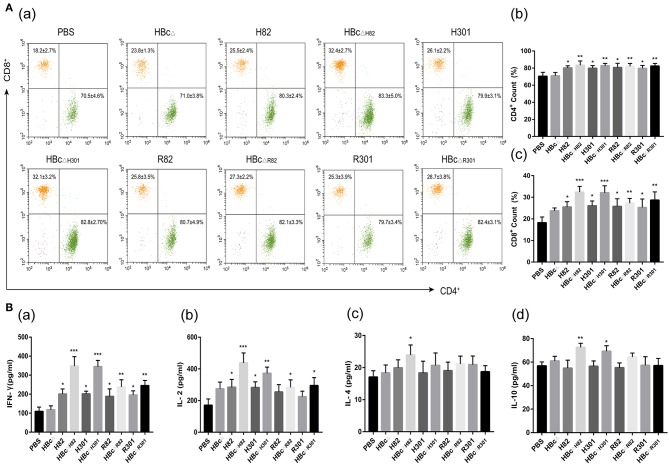
Cellular immune response induced by vaccination. Splenocytes were harvested from three mice per group 2 weeks after the final immunization. After stimulation with 10 μg/ml of the recombinant proteins, i.e., HBc_Δ_, H82, HBc_ΔH82_, H301, HBc_ΔH301_, R82, HBc_ΔR82_, R301, and HBc_ΔR301_, cellular immune responses were analyzed. **(A)** Percentages of T lymphocyte subsets in the immunized mice. After 72 h of stimulation, the lymphocytes were stained with anti-mouse CD3-APC, anti-mouse CD4-FITC and anti-mouse CD8-PE and were analyzed by flow cytometry. **(B)** Cytokine production in the immunized mice. The interleukin (IL)-2 and IL-4 levels in the supernatants of splenocytes at 24 h, IL-10 levels at 72 h, and interferon (IFN)-γ levels at 96 h were assessed with ELISA. Splenocytes from three mice in each group were tested individually, and the data represent mean ± SD values. *p < 0.05, **p < 0.01. ***p < 0.001.

After stimulation with 10 μg/ml of the recombinant proteins, i.e., HBc_Δ_, H82, HBc_ΔH82_, H301, HBc_ΔH301_, R82, HBc_ΔR82_, R301, or HBc_ΔR301_, or PBS alone (blank control), the percentages of CD4^+^ and CD8^+^ T lymphocytes subsets in the spleens of immunized mice were evaluated by flow cytometry 2 weeks after the third immunization. The percentages of CD4^+^ T cells in all the mice immunized with the recombinant proteins containing the CD4^+^ T cell epitope (AS15) (H82, HBc_ΔH82_, H301, HBc_ΔH301_, R82, HBc_ΔR82_, R301, and HBc_ΔR301_), especially the chimeric HBc particles (HBc_Δ*H*82_, HBc_Δ*H*301_, HBc_ΔR82_, and HBc_ΔR301_),were higher than those for the PBS group (*p* < 0.01; Figure 5Aa,b). Apart for the finding for the mice immunized with HBc_Δ_ and PBS, the percentages of CD8^+^ T cells in mice immunized with other recombinant proteins all increased, especially for the chimeric HBc VLPs containing the HF10 epitope (HBc_ΔH82_ and HBc_ΔH301_; *p* < 0.001; Figure 5Aa,c).

Splenocyte supernatants of the immunized mice were harvested at different times after restimulation with the recombinant proteins, conA or PBS to evaluate IL-2, IL-4, IL-10, and IFN-γ production by using ELISA. The IFN-γ levels in the splenocyte supernatants from the mice immunized with HBc_ΔH82_ and HBc_ΔH301_ (*p* < 0.001), HBc_ΔR82_ and HBc_ΔR301_ (*p* < 0.01), and with H82, H301, R82, and R301 (*p* < 0.05) proteins were significantly higher than those of the mice immunized with PBS (Figure 5Ba). The HBc_ΔH82_ (*p* < 0.001) and HBc_ΔH301_(*p* < 0.01) vaccinated mice had higher IL-2 levels than the control group (PBS) did (Figure 5Bb). However, a slight increase in IL-4 and IL-10 production was observed only in the mice immunized with HBc_ΔH82_ protein compared with those of PBS (Figure 5Bc,d). All these results indicated that the CD8^+^ T cell epitopes (HF10 and GRA7_20−28_) could both induce robust Th1-type cellular immune responses in mice. When these two peptides were loaded onto the carrier of HBc VLPs, the immune effect was more pronounced, especially for the HF10 peptide (HBc_ΔH82_ and HBc_ΔH301_). In addition, splenocytes from all groups of mice proliferated to comparable levels in response to ConA (Figure S1).

### Protection Against Acute and Chronic Toxoplasmosis

**Figure 6:**
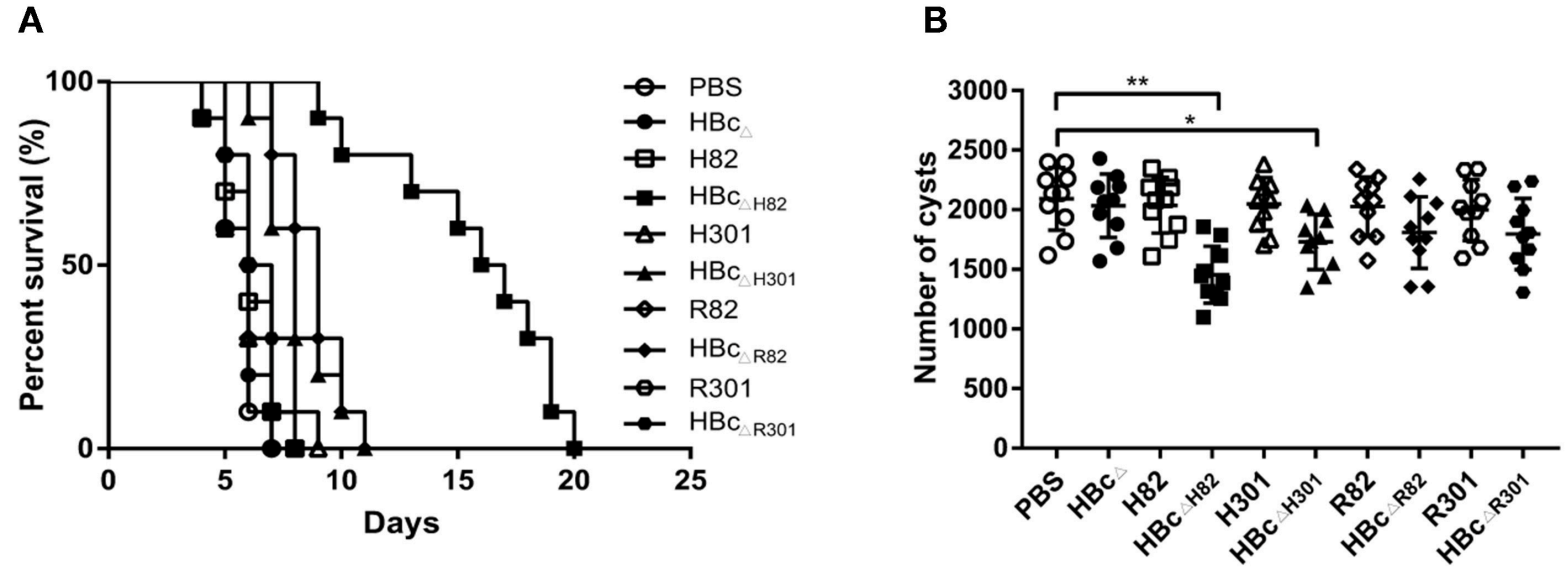
Protective experiments for acute and chronic toxoplasmosis. **(A)** Survival curve of the immunized mice after lethal challenge. Two weeks after the last immunization, 10 mice per group were intraperitoneally infected with 1 × 10^3^ tachyzoites of the *T. gondii* RH strain (type I) and observed daily for mortality. **(B)** Number of cysts in the brain after sublethal challenge. Another 10 mice per group were orally challenged with 30 cysts of the Pru strain (type II). Brain cysts were counted in surviving mice 45 days after the challenge. Data are representative results of three independent experiments and represent mean ± SD values. *p < 0.05, **p < 0.01.

To evaluate whether the recombinant proteins could induce effective protection against acute *T. gondii* infection, immunized mice were challenged with 1 × 10^3^ RH tachyzoites 2 weeks after the final immunization. The survival rates of the challenged mice are shown in Figure 6A. No significant difference was observed among the group immunized with PBS (5.6 ± 0.8 days) and those immunized with the recombinant proteins HBc_Δ_ (5.7 ± 0.9 days), H82 (6.1 ± 1.2 days), H301 (6.0 ± 1.4 days), HBc_ΔH301_ (8.1 ± 1.5 days), R82 (5.9 ± 1.2 days), HBc_ΔR82_ (8.8 ± 1.3 days), R301 (6.3 ± 1.1 days), and HBc_ΔR301_ (6.6 ± 1.2 days). The mice vaccinated with the chimeric HBc VLPs HBc_ΔH82_ (15.6 ± 3.8 days), HBc_ΔH301_(8.1 ± 1.5 days), HBc_ΔR82_(8.8 ± 1.3 days), and HBc_ΔR301_ (6.6 ± 1.2 days) were partially protected compared with the control group (PBS). The longest survival time was observed in the mice immunized with HBc_ΔH82_ protein, and one mouse even survived after 20 days of challenge.

The resistance provided by this vaccine formulation to *T. gondii* chronic infection was also evaluated by oral challenge the immunized mice with 30 Pru strain cysts. At 45 days after the challenge, the mice vaccinated with HBc_ΔH82_ (1454 ± 239; *p* < 0.01) and HBc_ΔH301_ (1730 ± 230; *p* < 0.05) proteins had fewer cysts than the PBS immunized mice did (2091 ± 263; Figure 6B). These results suggested that immunization of the mice with HBc_ΔH82_, which contained the conformational B cell epitopes (SAG1_82−102_), CD8^+^ T cell epitope (HF10), and CD4^+^ T cell epitope (AS15), induced substantial resistance to acute and chronic infection with *T. gondii*.

## Discussion

In this study, we inserted a B cell epitope (SAG1_82−102_ or SAG1_301−320_), a CD8^+^ cell epitope (HF10 or ROP7), and a CD4^+^ cell epitope (AS15) into truncated HBc_Δ_ (aa1–149) to construct four chimeric HBc VLPs, i.e., HBc_ΔH82_, HBc_ΔH301_, HBc_ΔR82_, and HBc_ΔR301_. When the chimeric HBc particles were expressed in *E. coli*, they showed icosahedral morphology similar to that of the original HBc protein. All these chimeric HBc VLPs induced strong humoral and cellular immune responses, along with high IgG antibody titer and IFN-γproduction. Prolonged survival time and decreased cyst burden in the brain were observed only in mice vaccinated with the HBc_ΔH82_ protein.

Hepatitis B virus (HBV) causes both acute and chronic infections of the liver (36). It is a small 42-nm virion carrying a viral DNA genome encapsulated within the HBV core antigen (HBcAg), which is surrounded by the lipid envelope with embedded HBV surface antigen (HBsAg) (10). HBsAg has been used as a VLP platform, but HBcAg has much higher immunogenicity than the surface antigen as a recombinant protein. It is increasingly being recognized that HBcAg-based VLPs are more efficient and flexible carrier moieties because of various characteristics (37). HBc particles can accommodate foreign antigen epitopes by presented them in three different ways: to the N-terminus or C-terminus, or inserted in the MIR of HBc; all these potential insertion sites could be very resilient in containing over 100 amino acids sequences of antigen without interference with the formation of capsids (6, 10). HBc consists of a protamine-like domain (aa150–183) that is dispensable for particle formation, and it typically contains RNA derived from the production host (e.g., *E. coli*), which is a dangerous component for vaccines used in humans and animals (9). Therefore, the truncated HBc (aa1-149) protein is more widely used as a VLP platform. HBc expressed by *E. coli* can spontaneously assemble into particles that are of two sizes, one with T = 3 icosahedral symmetry (90 dimers) and the other with T = 4 symmetry (120 dimers), and have a diameter of approximately 30 nm or 34 nm, respectively. The truncated HBc (aa1–149) forms ~ 95% of *T* = 4 capsids and ~ 5% of *T* = 3 capsids (35, 38). In our study, the truncated HBc (aa1–149) proteins containing amino acid sequences of antigen epitopes derived from *T. gondii* (HBc_ΔH82_, HBc_ΔH301_, HBc_ΔR82_, and HBc_ΔR301_) successfully assembled into icosahedral particles and were similar to the original non-chimeric HBc VLPs (HBc_Δ_). Most of these particles were uniform in size.

The MIR is located at the tips of the α-helical hairpins, which are known as surface-exposed “spikes” (around the codons for Asp78 and Pro79), on the HBc molecule (39). It is a traditionally used site for insertion of foreign epitopes and displays a high density of repetitive exogenous sequences; it is recognized by most of the antibodies induced by HBc particles to induce strong desirable cellular and humoral immune responses (30, 59). The C-terminal insertion (without the terminal protamine-like domain) also appears on the surface of assembled particles and is highly immunogenic (40). Therefore, in the current study, we inserted a B cell epitope (SAG1_82−102_ or SAG1_301−320_) and a CD8^+^ cell epitope (HF10 or ROP7) in the site between Asp^78^ and Pro^79^ and inserted a CD4^+^ cell epitope (AS15) into the C-terminus of the truncated HBc_Δ_(aa1–149) to construct the chimeric HBc VLPs. The malaria (*Plasmodium falciparum*) vaccine MalariVax (ICC-1132), produced by Apovia, has a similar foreign insertion site of MIR and the C terminal of HBc VLP and has already undergone Phase I clinical trials (41). In addition, all the chimeric HBc particles, i.e., HBc_ΔH82_, HBc_ΔH301_, HBc_ΔR82_, and HBc_ΔR301_, induced stronger humoral and cellular immune responses than their corresponding epitope peptide groups, i.e., H82, H301, R82, and R301, in the current study. This finding probably stems from the fact that peptides are extremely susceptible to enzymatic degradation and are poor immunogens on their own (42). HBc-VLP, which act as an excellent delivery system, could effectively protect them from degradation and greatly strengthen their immunogenicity. Furthermore, as a natural antigen, HBc particles act both as T-helper (Th) cell dependent and Th cell independent antigens, which allows HBc-VLPs to induce a strong adaptive immune response, characterized by high antibody titers and effective T-cell responses (10). To our knowledge, not much research has been performed on a *T. gondii* HBc-VLP vaccine. HBc VLPs are nanoparticles that can pass through tissue barriers and traffic to the lymph nodes more rapidly; they appear to be taken up more efficiently by professional antigen-presenting cells (APCs) (4). Nano-vaccine formulations that assemble GRA7-derived HLA-B*0702-restricted epitopes into nanospheres (SAPN) and load *T. gondii* protein extract in porous nanoparticles (DGNP) have been reported; all of these enhanced the immunogenicity of the antigens derived from the parasite (2, 31).

Antibodies play an important role in host immunity against *T. gondii* (43). Antigen-specific antibodies could directly block the tachyzoite in acute toxoplasmosis (44), and B cell-deficient mice are highly likely to develop inflammation in the brain in chronic toxoplasmosis (45). The antibody recognizes the specific part of the antigen, that is, the B cell epitopes, which are divided into two categories based on their structure and interaction with the antibody: conformational epitopes and linear epitopes (46). In the current study, the conformational B cell epitope (SAG1_82−102_) peptide (H82 and R82) did not induce higher IgG titers than PBS, but (SAG1_82−102_) did after insertion into the MIR of HBc (HBc_ΔH82_ and HBc_ΔR82_). These findings may be attributable to the fact that most B cell epitopes required to induce the desired humoral immune responses have to maintain their native conformation found in the protein (42);this is enabled by the MIR of HBc, which is composed of two antiparallel helices and forms the spikes of the particle (10, 39). In addition, we found that the conformational epitope SAG1_82−102_ elicited stronger antibody response than the linear epitope SAG1_301−320_ on the HBc-VLP platform. To our knowledge, conformational B cell epitopes have not been focused on *T. gondii* vaccine research to date.

Immunity to *T. gondii* is complex and involves many facets of the immune system. CD4^+^ T lymphocytes play an important role in shaping the immune responses and provide IL-2 for the development of CD8^+^ T lymphocytes, which can kill *T. gondii*-infected cells in an MHC-restricted, perforin-dependent manner and, more importantly, produce IFN-γ. IFN-γ helps to activate phagocytes to limit the propagation and spreading of the parasite by preventing the growth of tachyzoites and the reactivation of cysts. Therefore, it is critical for controlling both acute and chronic phases of *T. gondii* infection and its level determines the fate of the infection (34, 47–50). Thus, for a vaccine to mimic natural immunity, it should ideally comprise proteins or epitopes that can facilitate MHC class I processing and CD8^+^ T cells development. In the murine model of chronic toxoplasmosis, the resistance mediated by CD8^+^ T lymphocytes has been definitively mapped to the MHC class I *L*^*d*^ allele (11). To our knowledge, however, very few *T. gondii* specific *L*^*d*^-restricted CD8^+^ T cell epitopes have been reported to date; of these, only GRA6 (HPGSVNEFDF) (HF10) and ROP7 (IPAAAGRFF) epitopes can elicit robust T cell responses during the chronic phase (1, 13). GRA6 is a dense granule protein and is detected in the parasitophorous vacuole (PV) associated with the membranous network (51). ROP7 is a rhoptry bulb protein and is injected into the host cell cytoplasm relocalizing to the PV membrane, possibly interacting with the host cell cytoplasm (52). It is speculated that these two proteins, after secretion from the apical organelles (dense granules or rhoptries), are proteolytically cleaved into peptides in the proteosome of the infected cells, which are then presented on MHC *L*^*d*^ molecules to cytotoxic CD8^+^ T cells (13). Consistent with this, the current study showed that HF10 and ROP7 peptides both elicited a potent Th1 immune response, characterized by the activation of CD8^+^ T lymphocytes and increased production of IFN-γ and IL-2, especially the HF10 epitope on the HBc VLP platform (HBc_ΔH82_ and HBc_ΔH301_). In addition, the peptide AS15, which was presented by the A^b^ MHC class II molecule to the CD4^+^ T lymphocytes (20), successfully induced the development of protective CD4^+^ T cells in our study.

Most *T. gondii* strains can be broadly classified into three clonal lineages, which differ in their virulence. Type I strains (e.g., RH) are highly virulent in mice and are used in survival-based acute infection models. Low-virulence strains (e.g., type II Pru) have high cyst-forming ability in mice and are used in chronic infection models (11, 53). The survival rate and brain cyst burden of immunized mice are considered direct approaches for evaluating the protective efficacy of a candidate vaccine against acute and chronic toxoplasmosis (45, 54). In the current study, the mice immunized with HBc_ΔH82_ showed prolonged survival time and lesser brain cysts than mice immunized with PBS. These results are consistent with the above findings that only the mice immunized with HBc_ΔH82_ successfully triggered both strong humoral and cellular immune responses in all the vaccinated mice, and these two immune responses were interrelated and acted synergistically to increase protection against acute and chronic infection with *T. gondii* (49). Increased production of the cytokines IL-10 and IL-4, apart from IFN-γ and IL-2, may also be helpful against acute toxoplasmosis in immunized mice, because they can reduce short-term fatality by decreasing excessive and lethal Th1-type responses at the early acute stage of the disease (32, 55). A possible explanation is the activation of CD4^+^ T cells induced by AS15. Although most of them differentiate into Th1 cells triggered by IFN-γ secreted from the activated CD8^+^ T cells, a small number of CD4^+^ T cells still differentiate into TH2 cells, resulting in a slight but significant increase in IL-10 and IL-4 secretion. Similar activation of a T helper immune response with high levels of Th1 and low levels of Th2 related cytokines has been elicited by many other promising *T. gondii* vaccines also (33, 56, 57). In addition, decrease in the number of brain cysts was also observed in mice after vaccination with HBc_ΔH301_ protein, which contains the same peptide HF10 as HBc_ΔH82_. This may indicate that the useful features of HF10, as an immunodominant *L*^*d*^ gene restricted epitope whose region was found to control cyst formation, can be utilized well on this VLP carrier platform. Immunization of *L*^*d*^ mice with an HF10-containing vaccine comprising palmitic acid moieties or a monophosphoryl lipid A derivative was found to induce potent IFN-γ production from *L*^*d*^-restricted CD8^+^ T cells and protect mice from Pru strain challenge (11).

In conclusion, the HBc VLPs displayed a high density of effective antigenic epitopes of *T. gondii* on their surface and elicited potent humoral and cellular responses. The mice vaccinated with the HBc_ΔH82_ formulation survived after lethal and sublethal doses of parasite challenge. To our knowledge, no VLP vaccine against *T. gondii* that simultaneously contains a conformational B epitope, an *L*^*d*^-restricted CD8^+^ T cell epitope, and an *A*^*b*^-restricted CD4^+^ T cell epitope has been reported yet. Our study provides a novel and efficient strategy to develop vaccines against infection with this parasite. However, the significant differences between mice and humans, including those in MHC molecules, should not be ignored. Therefore, finding proteins or epitopes that are immunogenic and protective to the human body is an important topic for future research on vaccines against *T. gondii*; this also requires in-depth understanding of the molecular mechanisms governing the activation of the immune system in humans.

## Author Contributions

JG, SH, and HC designed experiments. JG, XS, and WS performed experiments and analyzed data. JG wrote the manuscript. AZ, HC, and CZ revised the manuscript. KA, GP, and HZ provided advice for experiment. All authors read and approved the final manuscript.

### Conflict of Interest Statement

The authors declare that the research was conducted in the absence of any commercial or financial relationships that could be construed as a potential conflict of interest.

## Supplementary Material

Figure S1 The production of cytokines in the immunized mice stimulated with ConA (positive control) at different times. The levels of IFN-γ **(A)**, IL-2 **(B)**, IL-4 **(C)**, and IL-10 **(D)** in the supernatants of splenocytes were assessed by ELISA.
