# Supplementary figures and images for: Immunogenicity of a Virus-Like-Particle Vaccine Containing Multiple Antigenic Epitopes of Toxoplasma gondii Against Acute and Chronic Toxoplasmosis in Mice
Source: Front Immunol. 2019 Mar 29;10:592. doi: 10.3389/fimmu.2019.00592 (PMC6449433; doi:10.3389/fimmu.2019.00592)

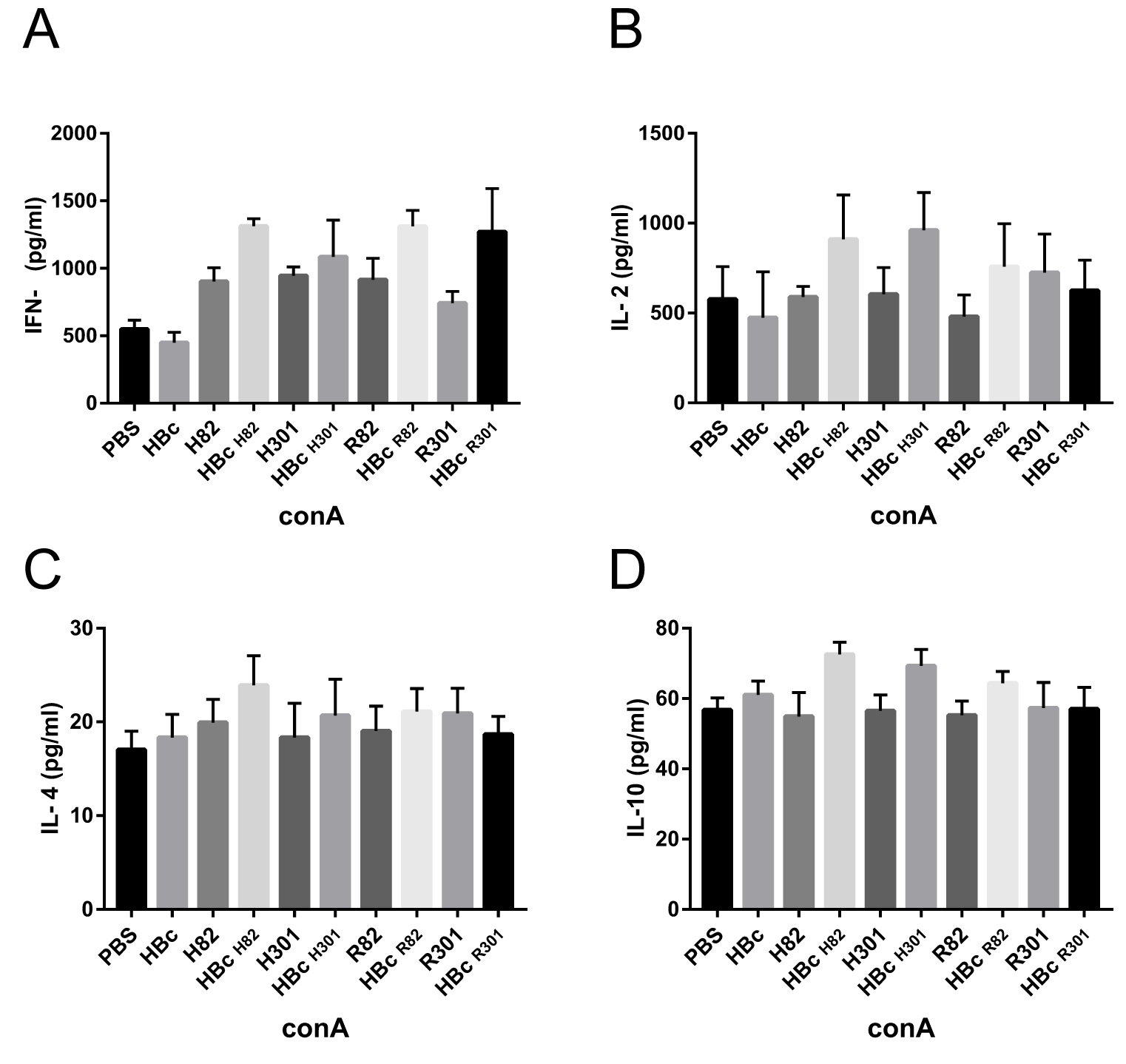

Supplement: Figure S1 — The production of cytokines in the immunized mice stimulated with ConA (positive control) at different times. The levels of IFN-γ (A), IL-2 (B), IL-4 (C), and IL-10 (D) in the supernatants of splenocytes were assessed by ELISA. [file Image_1.TIF]
